# Barriers to enrolment into a professional upgrading programme for enrolled nurses in Kenya

**Published:** 2012-12-26

**Authors:** Alice Lakati, Peter Ngatia, Caroline Mbindyo, Diana Mukami, Elizabeth Oywer

**Affiliations:** 1 Directorate of Capacity Building, AMREF, Kenya; 2 Nursing Council of Kenya, Kenya

**Keywords:** Nurses, enrolment, barriers, enrolment, training program, Curriculum

## Abstract

**Introduction:**

Nurses play a key role in the provision of health care. Over 70% of the nurses in Kenya are Enrolled Community Health Nurses (ECHNs). AMREF in partnership with Nursing Council of Kenya and the Ministry of Health pioneered an eLearning Nurse Upgrading Programme. The purpose of this study was to identify barriers that hindered enrolment into the programme.

**Methods:**

A descriptive cross-sectional design was used. A sample of 532 ECHNs was interviewed from four provinces. Data was collected using a pre-tested self administered questionnaire. Analysis was done using SPSS computer software. Descriptive statistics were calculated for all variables and chi-square tests used to determine variables that were associated with enrolment. Mann Whitney U-test was used for continuous variables.

**Results:**

A third (29.7%) of the nurses were from Rift Valley province and 17.9% from Coast. Majority (75%) were from public health facilities. The mean age of the nurses was 40.6 years. The average monthly income was KES 22,497.68 (USD 294). Awareness of the upgrading programme was high (97%) among the nurses. The cost of fees was the main (74.1%) barrier to enrolment in all the provinces and across all the health facilities. The type of health facility was significantly (p < 0.05) associated with enrolment. Nurses from faith-based health facilities were less likely to have enrolled.

**Conclusion:**

Awareness of the upgrading programme is high. The cost of upgrading programme, age and working in a faith-based health facility are the main barriers to enrolment. Intervention that fund nurses to upgrade would increase nurse enrolment.

## Introduction

Nurses are a significant component of the health workforce in Sub-Saharan Africa; probably more than in other continents. They constitute 45 - 60 percent of the entire health workforce and are responsible for a broad range of services [1]. There are several cadres of nurses based on their training. Enrolled nurses in Kenya are certificate holders trained for two and a half years. They work at operational levels in hospitals, health centres, dispensaries and communities. For many years they have worked in and managed dispensaries [2, 3]. According to a currently study, which refers to current statistic on nurse deployment in Kenya [2], registered community health nurses have been deployed to take charge of nursing services in health centres and, in some cases, dispensaries This situation necessitated the need to upgrade the skills of enrolled nurses to allow them gain more competencies that will lead to better service provision at all levels.

The Nursing Council of Kenya and the then Ministry of Health recognised the challenges the enrolled nurse skills poses to provision of quality health care and achievement of health-related MDGs. Therefore they collaborated with AMREF, to develop a print-based distance learning (DL) diploma programme that was launched in 2004. Although successful, this mode of learning proved relatively expensive due to the cost or printing, communication, and consistent review required by nursing curriculum. A different mode of training was needed. Therefore, the Nursing Council (NCK) adopted distance education after the realisation that the existing capacity for residential training would take 200 years to upgrade all the enrolled nurses to diploma level. Distance education was favoured because it would allow the nurses to learn at their pace and time without disrupting the vital services they provide in the communities. With the use of distance education, the NCK hoped to upgrade all the nurses in 5 years [4].

The introduction of the upgrading programme using a blended mode of both print and eLearning, witnessed a low enrolment than expected. By the end of 2004, only 34% of the 16,090 enrolled nurses had enrolled into the programme [4]. The main objective of this study was therefore to assess the barriers to enrolment among enrolled nurses in Kenya and provide recommendation on increasing enrolment. Upgrading the skills of nurses while at work will also reduce the critical shortage of human resources particularly in rural areas where majority of the enrolled nurses work.

## Methods

### Study design

A descriptive comparative cross-sectional design was used.

### Study population

Enrolled nurses in Kenya formed the study population. The focus of this study was the 10,600 ECHNs who according to NCK statistics, have not yet taken up upgrading. However, a smaller sample nurses who had already enrolled into the upgrading programme were also included to form a comparisons group. This group was specifically necessary to compare demographic characteristics between the nurses who have taken up upgrading and those who have not. This will lead to a better understanding of the barriers and increase validity of these study findings.

### Sampling methods

Epi Info 3.3.2 was used to determine the sample size of 564 nurses at 95% confidence interval and an expected frequency of 34% from a population of 10,600 enrolled nurses to achieve precision. A comparison was calculated from a population of 5,448 nurses who according to NCK had taken up upgrading using 80% confidence interval and an expected frequency of 50%. Therefore a sample of 159 was required to provide a comparison group. This sample was proportionately allocated to the four provinces. During data collection only 133 were interviewed based on their availability.

Four (4) provinces out of the eight (8) provinces in Kenya, were sampled to represent eight provinces in Kenya. The selection was done purposively with the knowledge of the demographic, economic and type of infrastructure in the province. Nyanza Province was selected as it has similar characteristics with Western Province and therefore Western Province was excluded. Eastern Province was selected as it has nearly similar characteristics with parts of North Eastern Province, therefore North Eastern was excluded. Coast Province was selected for it location and some parts of coast have similar characteristics with North Eastern province. This ensured that North Eastern is fully represented. Rift Valley Province was included as it is the largest province and has a big proportion of health facilities. Nairobi Province was selected for pre-testing.

A stratified sampling technique was used to determine the number of nurses to be interviewed allocated per province based on the number of nurses in that province. The provinces formed the strata. Once the sample per provinces was allocated, the list of all the health facilities per province was used to determine the number of nurses per each facility. This method allowed inclusion of nurses who meet the study criteria. Only nurses who were working during the day were interviewed. However, this does not bring bias because nurses work in shift rotation and therefore the interviewed nurses represent the enrolled nurses population.

### Data collection procedures and techniques

Research assistants were carefully selected and trained for 2 days on use of the data collection tool, selection of study subjects and adherence to completeness and accuracy. Supervisors who were nurse tutors were also trained and pre-testing was done in selected health facilities of Nairobi province.

A detailed structured self-administered questionnaire was used to collect data. This questionnaire contained questions on background information, socio- economic characteristics, programme awareness and barriers to enrolment. It was distributed to the nurses who met the inclusion criteria and they were given time to fill in the questionnaire. The research assistants assisted where necessary to ensure completeness.

### Data analysis methods

Four hundred and ninety (490) questionnaires from the study group and 133 in the control group were coded and entered into a computer using SPSS version 17.0. Data cleaning was done by running frequencies and all inconsistencies and errors in data entry were corrected. Two questionnaires out of 490 were incomplete or had inconsistent information. Therefore only 488 were analysed. Descriptive statistics were obtained in terms of frequencies and proportions for all categorical variables; and measures of central tendency and dispersions were used for continuous variables.

Chi-square tests were used to determine variables that significantly affect enrolment. Mann Whitney U-test and Kruskal Wallis test were used to determine significant differences in income across the provinces and type of health facility.

### Ethical considerations

The proposal was discussed and recommended for research submission by Directorate of Capacity Building - AMREF and Directorate of Health Systems Research and Policy - AMREF. The proposal then was submitted to and approved by the Kenya National Science council which awarded a research permit. Approval was also granted by the Ministry of Health, the Chief Nursing Officer, the Nursing Council and the Nursing Officers in Charge in each of the health facilities visited. A signed informed consent was obtained before the interview from all the nurses who participated in the study.

### Limitations

The desired sample size of 564 for the study group and 159 for the comparison group could not be attained. This was attributed to inaccurate records of the actual number of nurses per facility and the shift mode that nurses work. Only nurses who were on duty during the day were interviewed. However it this does not significantly affect the results since, the nurses on night shift are not different from those on day shift as they all work on rotation. During data collection it was clearly noted that not all the nurses could differentiate different modes of distance learning.

## Results

A total of 490 ECHNs who had not enrolled into the upgrading programme were interviewed from. Majority (74.8%) were from public health facilities and few (7.4%) were from private facilities. A larger proportion (83.3%) were female nurses and 78% married. The average age of the nurses was 40.6 years (95% CI= 39.8 - 41.4) and there were significant (p < 0.05) differences in age between nurses from private, public and faith-based health facilities. Public health facilities had significantly (p < 0.05) older nurses than the private or faith-based facilities. Nearly all (92.6%) of the nurses had attained secondary education, either KCE (20.9%) or KCSE (51.7%), and almost two thirds (74%) were employed on a permanent basis. Table 1 presents the background characteristics across provinces.

**Table 1:** Background characteristics of the study population

	Rift Valley (n=149)	Coast (n=86)	Nyanza (n=97)	Eastern (n=156)	Total (n=488)
**Facility Type**					
**Private Health Facility**	17.4%	1.2%	0%	5.8%	7.4%
**Public Health Facility**	56.4%	88.4%	93.8%	73.1%	74.8%
**Faith-based Health Facility**	26.2%	10.5%	6.2%	21.2%	17.8%
**Gender of Respondent**					
**Male**	11.3%	25.6%	18.4%	16%	16.7%
**Female**	88.7%	74.4%	81.6%	84%	83.3%
Age Group (Years)**					
**22-29**	16%	11.6%	23.7%	9.6%	14.7%
**30-39**	23.3%	36%	35.1%	25.6%	27.9%
**40-49**	43.3%	25.6%	36.1%	41.%	38.8%
**50+**	17.3%	26.7%	5.2%	23.7%	18.6%
Mean age**	41	41	37	42	41
**Marital Status**					
**Single**	12.2%	16.3%	11.2%	12.8%	12.9%
**Married**	72.8%	80.2%	79.6%	81.4%	78.3%
**Others (nun, widow, separated & divorced)**	15.0%	3.5%	9.2%	5.8%	8.8%
Education Level *					
**KAPE**	10%	9.6%	1%	7.7%	7.4%
**KCE**	18.7%	18.1%	26.6%	21.2%	20.9%
**KCSE**	56%	45.8%	60.3%	45.5%	51.8%
**EACE**	15.3%	26.5%	12.1%	25.6%	19.9%
**Employment Status**					
**Temporary/contract**	21.3%	23.3%	33.7%	28.2%	26.3%
**Permanent**	77.4%	76.7%	66.3%	71.2%	73.1%
**Volunteer(3)**	1.3%	0%	0%	0.6%	0.6%
**Number of Biological Children**					
**Median**	3	3	3	3	3
**Maximum**	9	8	7	8	9

** Significant differences across provinces p < 0.01

* Significant differences across provinces p < 0.05

The average income was found to be KES 22,323 with the lowest paid nurses earning KES 1,000 and the highest paid nurse earning KES 52,268. The average income did not differ significantly (p < 0.05) across provinces. However, there were significant (p < 0.05) differences across health facilities. Nurses from private and faith-based hospitals had a significantly lower income per month compared to nurses from the public sector (Table 2).

**Table 2:** Average income in Kshs in the four provinces and per facility type

VARIABLE	Average Income (Ksh)	Minimum Income (Ksh)	Maximum Income (Ksh)
**Province**			
**Rift Valley**	22,838	1,000	52,268
**Coast**	22,840	5,000	50,000
**Nyanza**	22,053	9,000	50,000
**Eastern**	21,747	7,000	36,502
**Total**	22,323	1,000	52,268
Facility**			
**Private**	19,559	6,000	52,000
**Public**	24,491	1,000	52,268
**Faith Based**	14,391	7,000	30,000
**Total**	22,323	1,000	52,268

** Kruskal Wallis p < 0.01, significant differences

Awareness of the Nurse Upgrading Programme in the study group was found to be high (97%), across all provinces. The classroom-based mode of study was the most (75.7%) well known across all facilities and half (51.3%) reported that they knew about the distance learning programme.

Colleagues were the most commonly (63.6%) reported source of information and the Nursing Council was the least commonly (25.7%) reported source of information on the Nurse Upgrading Programme (Table 3). Awareness level was found to be high across all the health facility types with colleagues remaining as the main source of information.

**Table 3:** Awareness of the upgrading Programme per province

VARIABLE	PROVINCE				
	Rift Valley	Coast	Nyanza	Eastern	Total
**Awareness**					
**Aware of upgrading programme**	96%	96.4%	100%	95.9%	97%
**Aware of Regular Programme**	77%	66.7%	80.8%	78.1%	75.7%
**Aware of distance learning**	52.7%	51.9%	50%	50%	51.3%
**Aware of eLearning**	71.4%	76.9%	72%	75%	73.9%
**Source of information**					
**Newspaper**	39.6%	43.4%	53.8%	33.7%	39.6%
**NCK**	29.7%	30.2%	32%	17.7%	25.7%
**Nursing school**	33%	37%	61.5%	37.5%	38.2%
**Colleagues**	61.5%	73.6%	69.2%	58.5%	63.6%
Nursing officer I/C*	45.1%	55.8%	23.1%	31.3%	36.6%
**Conference**	13.3%	20.4%	7.7%	10.4%	13.2%
**Learn from AMREF**	14.3%	7.4%	15.4%	15.6%	13.5%

* X^2^= 9.81 df=3 p < 0.05

Nearly all (96.3%) of the nurses in the study group reported that they are interested in enrolling in the Nurse Upgrading Programme. The type of information required about the Programme was similar (p > 0.05) across the four provinces. The cost of the course was the most (77.7%) reported information required followed by the admission criteria (64.4%). This trend was similar across the facilities

**Figure 1:**
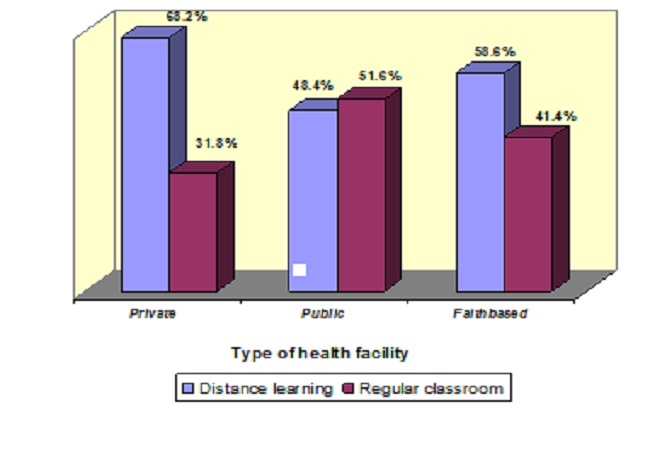
Preferred method of upgrading across different health facilities

Half (52.3%) of the nurses reported that they preferred distance learning and the rest preferred the regular classroom mode of upgrading. There were significant (p=0.017) difference in preferred mode of upgrading across the provinces with 63.4% of the nurses from Rift Valley Province preferring distance learning compared to 64% in Nyanza Province who preferred the classroom mode (Figure 1).

More than half (68.2%) of the nurses from private health facilities preferred distance learning, and slightly less than half (48.4%) of the nurses from public health facilities preferred distance learning. Although the proportions in preference show a difference, this difference was insignificant (p=0.451) indicating that the type of facility one is working in is unlikely to affect one's preference. Figure 1 shows the patterns of preference across facilities

Various reasons were given for the preference of classroom mode of upgrading. More than half (65.9%) of the nurses reported that; the classroom mode of upgrading allows one more time to read, while 40.6% indicated that, it provide an opportunity to be close to the tutors and a few (21%) reported that, it because they don't have computers.

Among the nurses who preferred distance learning, the main reason was convenience; 56% in private, 40% public and 42% faith-based health facilities. The problem of release from work station was significantly (p < 0.05) reported among the nurses working in the faith-based health facilities at 32.1% as compared to public health facilities at 14.7%. Table 4 shows the various reasons for preference of distance learning

**Table 4:** Reasons for preferring distance learning

REASON	FACILITY		
	Private	Public	Faith-based
Problems of release*	26.1%	14.7%	32.1%
**Completion speed**	21.7%	20%	10.7%
**Convenience reasons**	56.5%	40%	42%
**Finances reason**	21.7%	37.2%	32.1%
**Stress at work**	26.1%	30.1%	26.8%
**Cost of residential high**	4.3%	17.8%	11%

X^2^= 7.28 df=2 p < 0.05

### Barriers to enrolment

Almost three quarters (74.1%) of the nurses reported that the cost of school fees was the main barrier that has preventing them from enrolling in the Nurse Upgrading Programme. This finding showed no significant difference (p > 0.05) across all provinces. This is an indication that the cost of fees is a barrier across all the provinces. Another barrier linked closely to the cost of fees was having other financial commitments (50%). “I cannot get a loan” was reported more (36%) in Nyanza and Eastern (20.2%) province.

Analysis of the barriers across different health facilities (Table 5) indicated that the cost of fees and other financial commitments remain the major barrier; 77.4% and 44.4% respectively. Significant (p < 0.05) differences were however found whereby nurses from faith based facilities significantly reported that the fear of losing their job prevented them from enrolling in the Nurse Upgrading Programme. Another barrier was re-designation issues, which was. Re-designation was significantly (p=0.012) reported in public health facilities compared to faith-based and private.

**Table 5:** Barriers to enrolment per health facility

BARRIER	FACILITY		
	Private	Public	Faith-based
**Can't afford fees**	71.4%	73.5%	77.4%
**Can't get a loan**	14.3%	14.7%	22.2%
**Loan process burdensome**	4.8%	8.1%	11.1%
**Don't know how to enrol**	19%	10.8%	14.8%
Afraid to lose job**	19%	6.5%	22.2%
**Tried but never allowed**	9.5%	14.1%	9.3%
**Am about to retire**	9.5%	15.1%	2%
Have other financial commitments*	24%	54.6%	44.4%
Re-designation issues*	9.5%	13.5%	2%
**Experiences of other nurses**	9.5%	16.8%	5.6%
**Don't know admission criteria**	4.8%	5.9%	14.8%

* Chi-Square tests p < 0.05 Significant difference

** p < 0.01 Significant difference

Age was found to be significantly related to enrolment. Comparison between the control group and the study group showed that nurses who are already enrolled were significantly p=0.000) younger than those who have not enrolled. The average age for nurses who have enrolled was 38 years while those who have not enrolled had an average age of 41 years.

The type of health facility a nurse is working in was significantly related to enrolment. Among the ECHNs who had already enrolled, 38.6% were from public, 25.8% from private and 23.7% from Faith-based. This differences were found to be significantly (p=0.021). This shows that nurses from public health facilities were more likely to have enrolled compared to nurses from private and faith-based health facilities.

The level of education one had attained before training as a nurse was found to be significantly (p < 0.01) associated with enrolment. Majority (89%) of the nurses who had not attained secondary school education had not enrolled into the Programme. The study found that this group was also significantly (p < 0.05) older.

## Discussion

It is evident that nurses in Kenya are aware of the nurse upgrading programme and have interest in upgrading their skills to registered level. Our study has shown that the cost of school fees is a major barrier. This is further supported by the finding that income per month does not exceed USD 300. A major strength of the study is the coverage of 6 provinces in Kenya and all levels of health facilities where enrolled nurses work. The findings of this study are reliable in providing NCK and other partners in the nursing profession relevant information in addressing the barriers to enrolment into the upgrading programme in Kenya. It is believed that other sub-Saharan African countries with similar settings will find these study findings useful in their nursing programmes. One limitation of this study is its cross-sectional design, which could underestimate or over estimate the variable of interest at a point in time [5].

There are a few available studies that compare favourably with this study. However, it has been documented, that some major factors contributing to nurse migration from sub Sahara Africa SSA to western countries include economic/financial reasons (better pay) and opportunities for further education [6]. Economic factors not only contribute to out-migration but also hinder upgrading that leads to career progression. Similarly, Lack of funding for further education was a barrier to career progression among nurse practitioners in Victoria Australia. The cost among other factors was also reported as a barrier to continued professional development among health professionals and students through eLearning by Child et al [8] in a study conducted in Canada. Childs et al [8] also reported that despite the barriers that include cost, health professionals reported that eLearning, (a form of distance learning) was an effective method of education [7]. Similarly our study found half (52%) of the nurses preferring to upgrade through eLearning for reasons of convince.

Age, type of health facility (public compared to private and faith-based) and previous education were also found as significant (p ≤ 0.05) barriers to enrolling into the upgrading programme. Nurses working in faith-based and private facility were less likely to enrol and report reasons including fear of losing their job. Nurses from public health facilities were more likely to report reasons such as “am about to retire”, and re-designation issue. Re-designation refers to a situation whereby nurses who have upgraded to registered level are still designated as enrolled nurses at workplace. This situation is more common in public health facilities, a factor that de-motivates nurses into taking up upgrading. Of significant importance is the higher proportion of nurses from public health facilities that have enrolled into the upgrading programme compared to private and faith-based facilities. This indicates a government commitment in allowing nurses to take up upgrading. Furthermore, the ministries of health allow for study leave, a situation that is not clear in faith-based and private health facilities. These barriers can be compared to those reported in a similar study among rural and remote nurses in Canada [9], found that the barriers to nurses continued medical education were time constraint, family related reasons, financial constraint and heavy workload. Contrary to our findings, in the Canadian study, nurses who perceived barriers were more likely to be middle aged. It was not clear the actual definition of middle aged for the Canadian nurses, however, our study shows that older nurses (≥45 years) were less likely to enrol. Of practical interest to the Ministry of Health and NCK, is the finding that, there were significant differences in the age of the ECHNs in the provinces with Nyanza province having significantly younger nurses. This could be related to retirement or deployment of nurses in various provinces.

## Conclusion

This study concludes that awareness of the nurse upgrading programme is high. Nurses from all the four provinces and different levels of health facilities are aware of the Nurse Upgrading Programme. Enrolled nurses are interested in upgrading however, the cost of the upgrading programme is the main barrier to enrolment among all nurses. Working for a faith-based health facility hinders nurses from upgrading. Nurses from these facilities significantly feared losing their job and therefore choose not to enrol into the Programme. Age and nurse previous level of education are significant barriers to enrolment. Younger nurses and those who had attained secondary education are more likely to take up upgrading.

It is recommended that the Nursing Council of Kenya and other partners look for interventions that fund upgrading on nurses and establish working modalities for nurses in private and faith-based health facilities that will enable nurses working in this important institutions access upgrading.
